# Supplementary material for: From sequence to enzyme mechanism using multi-label machine learning
Source: BMC Bioinformatics. 2014 May 19;15:150. doi: 10.1186/1471-2105-15-150 (PMC4229970; doi:10.1186/1471-2105-15-150)
Supplement: Additional file 2 — Java code of ml2db. Additional file ml2db_code.tar.gz contains the Java source code to run the multi-label machine learning experiments and save the results to database. The code’s Javadoc is included. [file 1471-2105-15-150-S2.zip › additional file 2/ml2db/ml2db/doc/mulan/evaluation/measure/class-use/BipartitionMeasureBase.html]

Uses of Class mulan.evaluation.measure.BipartitionMeasureBase


JavaScript is disabled on your browser.


- Overview
- Package
- Class
- Use
- Tree
- Deprecated
- Index
- Help

- Prev
- Next

- Frames
- No Frames

- All Classes

## Uses of Class mulan.evaluation.measure.BipartitionMeasureBase

- Packages that use BipartitionMeasureBase

  | Package | Description |
  | --- | --- |
  |  |  |
  | --- | --- |
  | mulan.classifier.meta.thresholding |  |
  | mulan.evaluation.measure |  |
- - ### Uses of BipartitionMeasureBase in mulan.classifier.meta.thresholding

    Constructors in mulan.classifier.meta.thresholding with parameters of type BipartitionMeasureBase

    Constructor and Description

    `OneThreshold(MultiLabelLearner baseLearner,
    BipartitionMeasureBase aMeasure)`

    `OneThreshold(MultiLabelLearner baseLearner,
    BipartitionMeasureBase aMeasure,
    int someFolds)`

    `RCut(MultiLabelLearner baseLearner,
    BipartitionMeasureBase aMeasure)`

    Creates a new instance of RCut

    `RCut(MultiLabelLearner baseLearner,
    BipartitionMeasureBase aMeasure,
    int someFolds)`

    Creates a new instance of RCut

    `SCut(MultiLabelLearner baseLearner,
    BipartitionMeasureBase measure)`

    Creates a new instance of SCut

    `SCut(MultiLabelLearner baseLearner,
    BipartitionMeasureBase measure,
    int folds)`

    Constructor that initializes the learner with a base algorithm , Measure
    and num of folds
  - ### Uses of BipartitionMeasureBase in mulan.evaluation.measure

    Subclasses of BipartitionMeasureBase in mulan.evaluation.measure

    | Modifier and Type | Class and Description |
    | --- | --- |
    |  |  |
    | --- | --- |
    | `class` | `ExampleBasedAccuracy` Implementation of the example-based accuracy measure. |
    | `class` | `ExampleBasedBipartitionMeasureBase` Base class for example-based bipartition measures |
    | `class` | `ExampleBasedFMeasure` Implementation of the example-based F measure. |
    | `class` | `ExampleBasedPrecision` Implementation of the example-based precision measure. |
    | `class` | `ExampleBasedRecall` Implementation of the example-based recall measure. |
    | `class` | `ExampleBasedSpecificity` Implementation of the example-based recall measure. |
    | `class` | `HammingLoss` Implementation of the Hamming loss function. |
    | `class` | `HierarchicalLoss` Implementation of the Hierarchical loss measure. |
    | `class` | `LabelBasedBipartitionMeasureBase` Base class for label-based bipartition measures |
    | `class` | `LabelBasedFMeasure` Base implementation of the label-based macro/micro f-measures. |
    | `class` | `LabelBasedPrecision` Base implementation of the label-based macro/micro precision measures. |
    | `class` | `LabelBasedRecall` Common class for the micro/macro label-based recall measures. |
    | `class` | `LabelBasedSpecificity` Common class for the micro/macro label-based recall measures. |
    | `class` | `LossBasedBipartitionMeasureBase` |
    | `class` | `MacroFMeasure` Implementation of the macro-averaged f measure. |
    | `class` | `MacroPrecision` Implementation of the macro-averaged precision measure. |
    | `class` | `MacroRecall` Implementation of the macro-averaged recall measure. |
    | `class` | `MacroSpecificity` Implementation of the macro-averaged recall measure. |
    | `class` | `MicroFMeasure` Implementation of the micro-averaged precision measure. |
    | `class` | `MicroPrecision` Implementation of the micro-averaged precision measure. |
    | `class` | `MicroRecall` Implementation of the micro-averaged recall measure. |
    | `class` | `MicroSpecificity` Implementation of the micro-averaged recall measure. |
    | `class` | `SubsetAccuracy` Implementation of the subset accuracy measure. |

- Overview
- Package
- Class
- Use
- Tree
- Deprecated
- Index
- Help

- Prev
- Next

- Frames
- No Frames

- All Classes
